# Combined effect of mitochondrial DNA 5178 C/A polymorphism and alcohol consumption on estimated glomerular filtration rate in male Japanese health check-up examinees: a cross-sectional study

**DOI:** 10.1186/1471-2369-14-35

**Published:** 2013-02-12

**Authors:** Akatsuki Kokaze, Mamoru Ishikawa, Naomi Matsunaga, Kanae Karita, Masao Yoshida, Naoki Shimada, Tadahiro Ohtsu, Takako Shirasawa, Hirotaka Ochiai, Hiromi Hoshino, Yutaka Takashima

**Affiliations:** 1 Department of Public Health, Showa University School of Medicine, 1-5-8 Hatanodai, Shinagawa-ku, Tokyo, 142-8555, Japan; 2 Department of Public Health, Kyorin University School of Medicine, 6-20-2 Shinkawa, Mitaka-shi, Tokyo, 181-8611, Japan; 3 Mito Red Cross Hospital, 3-12-48 Sannomaru, Mito-shi, Ibaraki, 310-0011, Japan

**Keywords:** Alcohol, Chronic kidney disease, Estimated glomerular filtration rate, Mitochondrial DNA polymorphism

## Abstract

**Background:**

Prevention of chronic kidney disease (CKD) is a major public health issue. Although several studies have been performed on the association between alcohol consumption and CKD or renal function, it remains controversial. Numerous genetic polymorphisms have been reported to be associated with CKD and kidney function. Mitochondrial DNA cytosine/adenine (Mt5178 C/A) polymorphism is associated with longevity in Japanese. This polymorphism modifies the effects of alcohol consumption on blood pressure, risk of hypertension, serum triglyceride levels, risk of hyper-LDL cholesterolemia and serum uric acid levels. The objective of this study was to investigate whether Mt5178 C/A polymorphism modifies the effects of alcohol consumption on renal function in male Japanese health check-up examinees.

**Methods:**

A total of 394 male subjects aged 29–76 years were selected from among individuals visiting the hospital for regular medical check-ups. After Mt5178 C/A genotyping, a cross-sectional study assessing the combined effects of Mt5178 C/A polymorphism and habitual drinking on the risk of mildly decreased estimated glomerular filtration rate (eGFR) (<90 ml/min/1.73 m^2^) was conducted.

**Results:**

For Mt5178A genotypic men, habitual drinking may increase eGFR (*P* for trend = 0.003) or reduce the risk of mildly decreased eGFR (*P* for trend = 0.003). Daily drinkers had a significantly higher eGFR than non-drinkers (*P* = 0.005). The crude odds ratio for decreased eGFR was significantly lower in daily drinkers than in non-drinkers (odds ratio = 0.092, 95% confidence interval: 0.012-0.727, *P* = 0.024). On the other hand, for Mt5178C genotypic men, habitual drinking does not appear to affect eGFR.

**Conclusion:**

The present results suggest a joint effect of Mt5178 C/A polymorphism and alcohol consumption on eGFR and the risk of mildly decreased eGFR in male Japanese subjects.

## Background

Prevention of chronic kidney disease (CKD) is a major public health issue. Similarly to the survey reporting the prevalence of CKD in the United States [1], analysis of data, sampled from over half a million individuals in the general Japanese population, predicted that about 13% of the Japanese adult population had CKD in 2005 [2]. Considering that major outcomes of CKD are loss of kidney function and cardiovascular disease [3], primary prevention for CKD is required. Therefore, it is crucial to detect the modifiable risk factors of CKD using epidemiological approaches.

There have been several epidemiological studies on the association between alcohol consumption and CKD. A case–control study did not show an increased risk of CKD associated with alcohol consumption [4]. Another case–control study reported that consumption of more than two alcoholic drinks per day was associated with the risk of end-stage renal disease [5]. Both cross-sectional and longitudinal analyses in a population-based cohort showed an independent association between heavy alcohol consumption and CKD [6]. However, a large prospective cohort study suggested an inverse relationship between moderate alcohol consumption and the risk of renal dysfunction [7]. A large-scale cross-sectional study also showed an inverse association between frequency of alcohol consumption and CKD in healthy Japanese men [8]. These epidemiological studies did not assess genetic factors.

Mitochondrial DNA cytosine/adenine (Mt5178 C/A) polymorphism, which is also recognized as NADH dehydrogenase subunit-2 237 leucine/methionine (ND2-237 Leu/Met) polymorphism, is associated with longevity in Japanese [9]. The frequency of the Mt5178A genotype is significantly higher in Japanese centenarians than in the general population. Moreover, Japanese individuals with Mt5178A are more resistant to adult-onset diseases, such as hypertension [10], diabetes [11], myocardial infarction [12,13] and cerebrovascular disorders [14], than those with Mt5178C. This polymorphism modifies the effects of alcohol consumption on blood pressure [15], risk of hypertension [10], serum triglyceride levels [16], risk of hyper-LDL cholesterolemia [17] and serum uric acid levels [18]. In addition to these previous reports [10,15-18], several molecular epidemiological studies on CKD and kidney function [19-25] have encouraged us to examine the joint effects of Mt5178 C/A polymorphism and alcohol consumption on renal function.

In this study, we investigated whether longevity-associated Mt5178 C/A polymorphism modifies the effects of habitual alcohol consumption on renal function in male Japanese health check-up examinees.

## Methods

### Subjects

Participants were recruited from among individuals visiting the Mito Red Cross Hospital for regular medical check-ups between August 1999 and August 2000. This study was conducted in accordance with the Declaration of Helsinki and was approved by the Ethics Committee of the Kyorin University School of Medicine. Written informed consent was obtained from 602 volunteers before participation. Because the number of women was insufficient for classification into groups based on Mt5178 C/A genotype and alcohol consumption, women were excluded. Due to glomerular hyperfiltration in early diabetes [26], diabetic patients undergoing treatment were also excluded. Thus, 406 men were enrolled in the study. Twelve individuals with unclear data were subsequently excluded; therefore, subjects comprised 394 Japanese men aged 29–76 years.

### Clinical characteristics of subjects

Data on age, height, weight, blood pressure, serum lipid level, fasting plasma glucose level, serum uric acid level, blood urea nitrogen level and serum creatinine level were collected from the results of regular medical check-ups. Renal function was evaluated by estimated glomerular filtration rate (eGFR), which was calculated using a three-variable Japanese equation: eGFR = 194 × creatinine^− 1.094^ × age^− 0.287^[27]. Similarly to other genetic epidemiological studies [19,20], based on K/DOQI CKD classification [3], reduced eGFR was considered to be <90 ml/min/1.73 m^2^. Body mass index (BMI) was defined as the ratio of subject weight (kg) to the square of subject height (m). A survey of alcohol consumption, habitual smoking, coffee consumption, medical history and medication use was performed by means of questionnaire. Alcohol consumption was classified based on drinking frequency (daily drinkers; occasional drinkers, which include those who drink several times per week or per month; and non- or ex-drinkers). Smoking status was classified based on number of cigarettes smoked per day (never- or ex-smokers; 1–20 cigarettes smoked per day; and >20 cigarettes smoked per day). Coffee consumption was classified based on number of cups of coffee per day (≤1 cup per day; 2–3 cups per day; and ≥4 cups per day). For use of antihypertensive medication, subjects were classified as taking no drug treatment or taking medicine.

### Genotyping

DNA was extracted from white blood cells using the DNA Extractor WB kit (Wako Pure Chemical Industries, Osaka, Japan). The Mt5178 C/A polymorphism was detected by polymerase chain reaction (PCR) and digestion with *Alu*I restriction enzyme. The sequence of primers was: forward 5^′^-CTTAGCATACTCCTCAATTACCC-3^′^, reverse 5^′^-GTGAATTCTTCGATAATGGCCCA-3^′^. PCR was performed with 50 ng of genomic DNA in buffer containing each primer at 0.2 μmol/l, 1.25 mmol/l dNTPs, 1.5 mmol/l MgCl_2_, and 1 U of Taq DNA polymerase. After initial denaturation at 94°C for 5 min, PCR was conducted through 40 cycles as follows: denaturation at 94°C for 30 s, annealing at 60°C for 60 s and polymerase extension at 72°C for 90 s. After cycling, a final extension at 72°C for 10 min was performed. PCR products were digested with *Alu*I restriction enzyme (Nippon Gene, Tokyo, Japan) at 37°C overnight, and were electrophoresed on 1.5% agarose gels stained with ethidium bromide for visualization under ultraviolet light. The absence of an *Alu*I site was designated as Mt5178A, and the presence of this restriction site was designated as Mt5178C.

### Statistical analyses

Statistical analyses were performed using SAS statistical software, version 9.2 for Windows. Multiple logistic regression analysis was used to calculate odds ratios (OR) for the reduction of eGFR (<90 ml/min/1.73 m^2^). Covariates were selected for their ability to confound the associations as determined through univariate and stepwise models. For multiple logistic regression analysis and analysis of covariance, habitual smoking (never- or ex-smokers = 0; 1–20 cigarettes smoked per day = 1; >20 cigarettes smoked per day = 2), coffee consumption (≤1 cup per day = 1; 2–3 cups per day =2; ≥4 cups per day = 3) and antihypertensive medication use (no use of antihypertensive = 0; use of antihypertensive = 1) were numerically coded. Differences with P values of less than 0.05 were considered to be statistically significant.

## Results

No significant differences in age, BMI, systolic blood pressure, diastolic blood pressure, serum total cholesterol levels, serum HDL cholesterol levels, serum triglyceride levels, fasting plasma glucose levels, serum uric acid levels or blood urea nitrogen levels were observed between the Mt5178C and Mt5178A genotypes (Table 1). There were no outliers in eGFR for either genotype; eGFR in Mt5178C genotypic men ranged from 50 to 119 ml/min/1.73 m^2^ and that in Mt5178A genotypic men ranged from 52 to 112 ml/min/1.73 m^2^. Among men with Mt5178C, the frequency of daily drinkers was 46.4%, that of occasional drinkers was 35.2% and that of non-drinkers was 18.4%. Among those with Mt5178A, the frequency of daily drinkers was 47.7%, that of occasional drinkers was 38.7% and that of non-drinkers was 13.6%. Chi-squared test showed no significant differences in alcohol consumption between the Mt5178 C/A genotypes (*P* = 0.426).

**Table 1:** Clinical characteristics of study subjects by Mt5178 C/A genotype

	**Mt5178C**	**Mt5178A**	***P*value**
	**N = 239**	**N = 155**	
Age (years)	54.3 ± 7.8	53.2 ± 7.8	0.171
Body mass index (kg/m^2^)	23.3 ± 2.8	23.5 ± 2.6	0.461
Systolic blood pressure (mmHg)	125.8 ± 15.9	125.7 ± 14.1	0.940
Diastolic blood pressure (mmHg)	74.0 ± 10.7	73.8 ± 9.1	0.823
Serum total cholesterol (mg/dl)	203.5 ± 34.3	202.1 ± 31.8	0.672
Serum HDL cholesterol (mg/dl)	54.6 ± 13.6	56.3 ± 16.2	0.269
Serum triglyceride (mg/dl)	136.7 ± 91.1	139.5 ± 90.8	0.766
Fasting plasma glucose (mg/dl)	97.2 ± 9.4	97.6 ± 9.7	0.672
Serum uric acid (mg/dl)	5.98 ± 1.21	5.93 ± 1.21	0.673
Blood urea nitrogen (mg/dl)	15.8 ± 3.7	15.2 ± 3.4	0.143
Serum creatinine (mg/dl)	0.817 ± 0.117	0.797 ± 0.111	0.090
eGFR (ml/min/1.73 m^2^)	79.1 ± 13.1	81.5 ± 12.4	0.066
Renal function (eGFR ≥ 90/90 > eGFR ≥ 60/eGFR < 60) (%)	17.6/77.8/4.6	25.2/72.2/2.6	0.133
Antihypertensive medications (%)	18.8	12.9	0.122
Alcohol consumption (daily drinkers/occasional drinkers/non-drinkers) (%)	46.4/35.2/18.4	47.7/38.7/13.6	0.426
Smoking status (never- or ex-/1–20 cigarettes per day/>20 cigarettes per day) (%)	59.4/29.7/10.9	59.4/25.8/14.8	0.429
Coffee consumption (≤1 cup per day/2–3 cups per day/≥4 cups per day) (%)	61.1/29.7/9.2	55.5/32.9/11.6	0.510

*HDL*; high-density lipoprotein. *eGFR*; estimated glomerular filtration rate. Age, body mass index, systolic blood pressure, diastolic blood pressure, serum total cholesterol levels, serum HDL cholesterol levels, serum triglyceride levels, fasting plasma glucose levels, serum uric acid levels, blood urea nitrogen levels, serum creatinine levels and eGFR are given as means ± S.D. For renal function, antihypertensive medications, alcohol consumption, smoking status, and coffee consumption, *P* values were calculated by chi-squared test. All *P* values indicate the significance of differences between Mt5178C and Mt5178A.

For Mt5178A genotypic men, the frequency of alcohol consumption was positively and significantly associated with eGFR (*P* for trend = 0.003) (Table 2). After adjustment, this positive association between alcohol consumption and eGFR remained significant. Moreover, eGFR was significantly higher in daily drinkers than in non-drinkers (*P* = 0.005). After adjustment for age and BMI or for age, BMI, habitual smoking, coffee consumption and use of antihypertensive medication, eGFR was also significantly higher in daily drinkers than in non-drinkers (*P* = 0.025 and *P* = 0.026, respectively). On the other hand, although eGFR was significantly higher in occasional drinkers with Mt5178C than in non-drinkers with Mt5178C (*P* = 0.043), the association between Mt5178C genotype and eGFR did not appear to depend on the frequency of alcohol intake.

**Table 2:** Estimated glomerular filtration rates by alcohol consumption and Mt5178 C/A genotype

**Genotype and alcohol consumption**	**eGFR (ml/min/1.73 m**^**2**^**)**	**eGFR † (ml/min/1.73 m**^**2**^**)**	**eGFR ‡ (ml/min/1.73 m**^**2**^**)**
Mt5178C (N = 239)			
Non-drinkers (N = 44)	75.8 ± 2.0	76.5 ± 1.9	74.5 ± 2.3
Occasional drinkers (N = 84)	81.8 ± 1.4*	81.1 ± 1.3	79.1 ± 1.9
Daily drinkers (N = 111)	78.4 ± 1.2	78.6 ± 1.2	76.8 ± 1.6
	*P* for trend = 0.667	*P* for trend = 0.693	*P* for trend = 0.612
Mt5178A (N = 155)			
Non-drinkers (N = 21)	74.3 ± 2.6	75.8 ± 2.6	75.8 ± 3.0
Occasional drinkers (N = 60)	81.3 ± 1.6	81.0 ± 1.5	80.8 ± 2.1
Daily drinkers (N = 74)	83.8 ± 1.4**	83.6 ± 1.4*	83.7 ± 2.0*
	*P* for trend = 0.003	*P* for trend = 0.009	*P* for trend = 0.008

*eGFR*; estimated glomerular filtration rate. †eGFR is given as least-square mean ± S.E. adjusted for age, body mass index; ‡eGFR is given as least-square mean ± S.E. adjusted for age, body mass index, habitual smoking, coffee consumption and antihypertensive medication use. Bonferroni correction for multiple comparisons was used. *P < 0.05 vs. non-drinkers, **P < 0.01 vs. non-drinkers.

For subjects with Mt5178A, the risk of decreased eGFR may depend on frequency of alcohol consumption (*P* for trend = 0.003) (Table 3). After adjustment, the negative association between increasing frequency of alcohol consumption and the risk of decreased eGFR remained significant. The crude OR for decreased eGFR was significantly lower in daily drinkers than in non-drinkers (OR = 0.092, 95% confidence interval (CI): 0.012–0.727, *P* = 0.024). After adjustment for age and BMI or for age, BMI, habitual alcohol consumption, coffee consumption and use of antihypertensive medication, a significant OR remained (adjusted OR = 0.098, 95% CI: 0.012–0.784, *P* = 0.029 and adjusted OR = 0.091, 95% CI: 0.011–0.747, *P* = 0.026, respectively). On the other hand, the association between Mt5178C genotype and the risk of decreased eGFR does not appear to depend on alcohol consumption.

**Table 3:** **Odds ratios (ORs) and 95% confidence intervals (CIs) for decreased eGFR (< 90 ml/min/1.73 m**^**2**^**) by Mt5178 C/A genotype and alcohol consumption**

**Genotype and alcohol consumption**	**Frequency (%)**		**OR (95% CI)**	**Adjusted OR† (95% CI)**	**Adjusted OR‡ (95% CI)**
	**eGFR ≥ 90 ml/min/1.73 m**^**2**^	**eGFR < 90 ml/min/1.73 m**^**2**^			
Mt5178C (N = 239)					
Non-drinkers	6 (13.6)	38 (86.4)	1 (reference)	1 (reference)	1 (reference)
Occasional drinkers	20 (23.8)	64 (76.2)	0.505 (0.186–1.369)	0.633 (0.222–1.810)	0.786 (0.261–2.372)
Daily drinkers	16 (14.4)	95 (85.6)	0.938 (0.341–2.576)	1.038 (0.359–2.999)	1.024 (0.339–3.092)
			*P* for trend = 0.690	*P* for trend = 0.587	*P* for trend = 0.587
Mt5178A (N = 155)					
Non-drinkers	1 (4.8)	20 (95.2)	1 (reference)	1 (reference)	1 (reference)
Occasional drinkers	12 (20.0)	48 (80.0)	0.200 (0.024–1.642)	0.276 (0.032–2.395)	0.280 (0.031–2.505)
Daily drinkers	26 (35.1)	48 (64.9)	0.092 (0.012–0.727)*	0.098 (0.012–0.784)*	0.091 (0.011–0.747)*
			*P* for trend = 0.003	*P* for trend = 0.004	*P* for trend = 0.004

*eGFR*; estimated glomerular filtration rate. †OR adjusted for age and body mass index. ‡OR adjusted for age, body mass index, habitual smoking, coffee consumption and antihypertensive medication. *P < 0.05.

## Discussion

In the present study, we observed that Mt5178 C/A polymorphism apparently modifies the effects of habitual alcohol drinking on eGFR in Japanese men. This is a new gene-environment interaction on renal function. For men with Mt5178A, habitual alcohol consumption may reduce the risk of mildly decreased eGFR. On the other hand, for those with Mt5178C, alcohol consumption does not appear to influence eGFR.

Schaeffner et al. reported an inverse association between moderate alcohol consumption and the subsequent risk of renal dysfunction in large cohort of apparently healthy men [7]. They used eGFR calculated by the Cockcroft-Gault equation, and reported that men who consumed at least seven drinks per week had an approximately 25% lower risk of reduced eGFR (<55 ml/min) in a 14-year period than those who consumed one or fewer drinks per week. Funakoshi et al. also revealed an inverse relationship between frequency of alcohol consumption and CKD in apparently healthy men [8]. They used eGFR calculated using the three-variable Japanese equation and found that everyday drinkers had an approximately 40% lower risk of CKD (eGFR <60 ml/min/1.73 m^2^) than non-drinkers. Judging from both investigations [7,8], alcohol consumption was observed to have a desirable effect on the risk of decreased eGFR. However, our observations suggest that genetic information is required to assess the relationship between alcohol intake and renal function.

From the viewpoint of preventing mildly decreased eGFR, habitual drinking appears to be beneficial for Mt5178A genotypic men. Moreover, for men with Mt5178A, alcohol consumption decreases serum triglyceride levels [16]. However, it is uncertain whether alcohol consumption is beneficial overall for Mt5178A genotypic men. In contrast, for Mt5178C genotypic men, daily alcohol consumption may increase the risks of hypertension [10] and hyperuricemia [18], but may also reduce the risk of hyper-LDL cholesterolemia [17]. Thus, it is unclear whether alcohol consumption is detrimental for Mt5178C genotypic men. Although further genetic epidemiological research is necessary, genotyping of Mt5178 C/A is thought to be practical for personalized prevention of lifestyle-related diseases, including CKD.

The mechanisms underlying the joint effects of Mt5178 C/A polymorphism and alcohol consumption on renal function have not been elucidated. This gene-environment interaction is presumed to result from differences in the biophysical and biochemical properties of ND2-237 Leu/Met. NADH dehydrogenase is regarded as the major physiological and pathological site of reactive oxygen species (ROS) generation in mitochondria, and as a target of assault by ROS [28]. Mouse mitochondrial DNA 4738 C/A (Mt4738 C/A) polymorphism leads to a leucine to methionine substitution in NADH dehydrogenase subunit 2. ROS production by NADH dehydrogenase is significantly lower in mice with Mt4738A than in those with Mt4738C [29]. The results in experimental mice indicate that ND2-237Met suppresses ROS production in humans. Moreover, as methionine residues play an antioxidant role in scavenging ROS [30], ND2-237Met may also protect NADH dehydrogenase itself from ROS attack.

Ethanol metabolism is directly involved in the production of ROS [31]. There may be biophysical and biochemical differences in the protection against ethanol-induced ROS or the reduction of ethanol-induced ROS generation between ND2-237Leu and ND2-237Met. These apparent disparities are thought to result in the combined effects of Mt5178 C/A polymorphism and habitual drinking on eGFR. Recently, using male C57BL/6 mice, Yuan et al. demonstrated that moderate ethanol exposure can provide protection for kidneys against ischemia/reperfusion-induced renal injury by enhancing antioxidant capacity characterized by higher activity of superoxide dismutase, which is a critical enzyme responsible for detoxifying ROS [32]. However, to determine the mechanisms responsible for interaction between ND2-237 Leu/Met genotypes and alcohol consumption on renal function, further biochemical and pharmacological studies are necessary.

In the Japanese population, several genetic polymorphisms have been reported to be associated with CKD, defined as eGFR <50 ml/min/1.73 m^2^[21,22] or <60 ml/min/1.73 m^2^[23,24]. In addition, promoter polymorphism of the endotoxin receptor [19], single nucleotide polymorphism rs1287637 in the nephronophthisis 4 gene is associated with mildly decreased eGFR (<90 ml/min/1.73 m^2^) [20]. Therefore, these molecular epidemiological studies, as well as our results, indicate gene-gene or gene-gene-environment interactions on renal function.

In accordance with K/DOQI CKD classification [3], eGFR of <90 ml/min/1.73 m^2^ was defined as reduced eGFR in this cross-sectional study. In the present subjects, the prevalence of eGFR of <90 ml/min/1.73 m^2^ was 82.4% in men with Mt5178C and was 74.8% in those with Mt5178A. A large-scale population-based epidemiological study showed that the prevalence of eGFR of <90 ml/min/1.73 m^2^ was 80.6% in Japanese men aged 30–69 years [2]. Therefore, whether the validity of the definition of reduced eGFR adopted in our study is worthy of further consideration.

There are several important limitations in this study. First, as compared with other genetic epidemiological studies on renal function [19-25], the study sample was too small. Second, selection bias is likely due to the recruiting of subjects from those visiting the hospital for regular medical check-ups, and the prevalence of moderately decreased eGFR of <60 ml/min/1.73 m^2^, recognized as CKD, was low among the study subjects. Third, this study was a cross-sectional study, and although the study design can suggest causal links, it cannot establish valid causality. To overcome these limitations, a large-scale population-based follow-up study is necessary. Fourth, because of lack of data on the amount of alcohol intake, the evaluation of habitual alcohol intake was based on the frequency of alcohol consumption. Although we have also used this evaluation method in previous studies [10,15-18], the existence of joint effects between Mt5178 C/A polymorphism and volume of alcohol intake on eGFR warrants further investigation. Finally, we lacked information on proteinuria, which is an early and sensitive marker of kidney damage in various types of CKD [3].

## Conclusion

This study identified a novel joint effect by a genetic factor and lifestyle behavior on renal function. Longevity-associated Mt5178 C/A polymorphism may modulate the effects of alcohol consumption on eGFR or the risk of mildly decreased eGFR in Japanese men. For Mt5178A genotypic men, habitual drinking may increase eGFR or reduce the risk of mildly decreased eGFR. On the other hand, for Mt5178C genotypic men, habitual drinking does not appear to affect eGFR. Together with a complete evaluation of the biophysical or biochemical effects of alcohol intake, genetic information on Mt5178 C/A polymorphism may contribute to primary individualized prevention of CKD and consequential reductions in either kidney dysfunction or cardiovascular disease.

## Competing interests

The authors declare that they have no competing interests.

## Authors’ contributions

AK designed the study, carried out the epidemiological survey, carried out the genotyping, analyzed the data, and drafted the manuscript; MI collected the samples; NM assisted with genotyping; KK and MY carried out the epidemiological survey; NS, TO, TS, HO and HH assisted in data analysis and interpretation; YT designed the study and carried out the epidemiological survey. All authors have read and approved the final manuscript.

## Pre-publication history

The pre-publication history for this paper can be accessed here:

http://www.biomedcentral.com/1471-2369/14/35/prepub
